# Antithrombin significantly influences platelet adhesion onto immobilized fibrinogen in an in-vitro system simulating low flow

**DOI:** 10.1186/1477-9560-4-19

**Published:** 2006-10-13

**Authors:** Robert Loncar, Uwe Kalina, Volker Stoldt, Volker Thomas, Rüdiger E Scharf, Aleksandar Vodovnik

**Affiliations:** 1 Department of Hemostasis and Transfusion Medicine, Heinrich Heine University Medical Center Duesseldorf, Germany; 2 Research ZLB Behring, Emil von Behring Strasse 76, 35041 Marburg, Germany; 3 Department of Histopathology, The Calderdale Royal Hospital, HX3 0PA Halifax, UK

## Abstract

**Background:**

Adhesion of platelets onto immobilized fibrinogen is of importance in initiation and development of thrombosis. According to a recent increase in evidence of a multiple biological property of antithrombin, we evaluated the influence of antithrombin on platelet adhesion onto immobilized fibrinogen using an in-vitro flow system.

**Methods:**

Platelets in anticoagulated whole blood (29 healthy blood donors) were labelled with fluorescence dye and perfused through a rectangular flow chamber (shear rates of 13 s^-1 ^to 1500 s^-1^). Platelet adhesion onto fibrinogen-coated slips was assessed using a fluorescence laser-scan microscope and compared to the plasma antithrombin activity. Additionally the effect of supraphysiological AT supplementation on platelets adhesion rate was evaluated.

**Results:**

Within a first minute of perfusion, an inverse correlation between platelet adhesion and plasma antithrombin were observed at 13 s^-1 ^and 50 s^-1 ^(r = -0.48 and r = -0.7, p < 0.05, respectively). Significant differences in platelet adhesion related to low (92 ± 3.3%) and high (117 ± 4.1%) antithrombin activity (1786 ± 516 U vs. 823 ± 331 U, p < 0.05) at low flow rate (13 s^-1^, within first minute) have been found. An in-vitro supplementation of whole blood with antithrombin increased the antithrombin activity up to 280% and platelet adhesion rate reached about 65% related to the adhesion rate in a non-supplemented blood (1.25 ± 0.17 vs. 1.95 ± 0.4 p = 0.008, respectively).

**Conclusion:**

It appears that antithrombin in a low flow system suppresses platelet adhesion onto immobilized fibrinogen independently from its antithrombin activity. A supraphysiological substitution of blood with antithrombin significantly reduces platelet adhesion rate. This inhibitory effect might be of clinical relevance.

## Background

The scope of the problem of arterial and venous thrombosis is staggering since at least 5 million adults in the United States alone suffer from related symptoms. About 50% of the annual non-accidental deaths in the United States are caused by thrombi predominantly composed of platelets in the coronary or cerebral arteries.

Antithrombin (AT), in the past also referred as antithrombin III, is a potent inhibitor of the coagulation cascade [1]. Although the name, antithrombin, implies that it works only on thrombin, it actually serves to inhibit virtually all of the coagulation enzymes to at least some extent. The primary enzymes it inhibits are factor Xa, factor IXa and thrombin (factor IIa). AT have also inhibitory actions on the factor XIIa, factor XIa, complex of factor VIIa and tissue factor. AT ability to limit coagulation through the multiple interactions makes it one of the primary natural anticoagulant proteins [1,2].

AT is a 58 kd single-chain α2-glycoprotein with plasma concentration of approximately 150 μg/mL. AT is composed of 432 amino acids to which four oligosaccharide chains are attached [3]. The inhibition of thrombin is due to forming a 1:1 stoichiometric complex between the two components via one reactive site (arginine)-active centre (serine) interaction. This relatively slow interaction is dramatically accelerated in the presence of heparin. Through the binding of heparin on the AT and consecutive change of AT configuration capability to neutralise clotting enzymes increases above the 1000 folds [1,4]. This interaction is also the basis for the use of heparin and low-molecular-weight heparin as medications as anticoagulants [4,5].

Once formed, the equimolar thrombin-antithrombin (TAT) complex is removed in the liver with one half-life of less than 5 minutes (half-life of native AT is about 60 hours) [1]. AT have multiple biological properties, which are in last few years in focus of interest of many researchers [5-12].

A rapid AT consumption was observed in different clinical entities [1,11]. Since decreased AT plasma concentration correlated with increased mortality in related clinical events, the concept of exogenous AT substitution has been established [11]. Conducted studies showed that a beneficial effect of AT in some clinical events may be due to both its anticoagulant activity and the other unknown activity [12]. In some extent clinical improvements were associated with significant modulation of interleukin-6 plasma levels and down regulation of intercellular adhesion molecule-1 (ICAM-1), as well as E-selectin [12,13]. It has been recently shown that AT causes a reduction of ischaemia-reperfusion induced damage of renal tissue [14]. This effect seems not be caused solely by inhibition of thrombin generation because selective inhibition of thrombin did not prove similar effect [8,14]. Hoffmann et al. [15] reported that AT reduces the endotoxin-induced leukocyte adherence onto vessel wall and improves capillary perfusion. Inhibitory effects of AT on leukocyte and platelet recruitment to the post ischaemic retina and consecutive protective property against ischaemia/reperfusion injury were also observed in vivo rat model [10,16]. However, up to day there is no information about the effects of AT on the platelet adhesion under various flow conditions. In this study we evaluated role of AT on the platelet adhesion onto immobilized fibrinogen under different shear stress conditions.

## Methods and subjects

### Subjects

Twenty-nine healthy blood donors from our blood bank were enrolled in this study. Mean age was 44 ± 12 years, 21 were men and eight were women. None of the donors had taken any medication in the preceding 14 days. Women on hormone replacement therapy and hormonal contraception, smokers, obese persons, persons with positive familiar anamnesis related to the arterial or venous vessel disease were excluded. Global coagulation tests, factors of coagulation and standard biochemical parameters were in the normal range according to the international standards. This study was performed according to the Helsinki declaration and was approved by the local ethical committee.

### Blood preparation

Venous blood obtained from the cubital vein was immediately anticoagulated with PPACK in final concentration of 40 μM. During incubation period (60 min at 37°C) platelets were labelled with fluorescence dye Mepacrine (quinacrine dihydrochloride final concentration of 10 μM; Sigma Chemical), which was immediately accumulated by delta granules of platelets [17]. The blood was perfused through rectangular flow chamber within 2 hours of withdrawal.

Effect of supraphysiological AT substitution onto platelet adhesion was tested in blood obtained from eight subjects. The collected blood was aliquoted and one aliquot was additionally incubated with AT (30 min, 2.8 IU/ml of blood, Kybernin 500, Aventis, Marburg, Germany) prior to perfusion through the flow chamber. In control experiments the same volume of 0.9% NaCl was added to blood prior to perfusion. Blood obtained from eight subjects were anticoagulated with PPACK and blood obtained from four donors were anticoagulated with heparin (17/IU/ml, Liquemin N, Hoffmann-La Roche AG, Grenzach-Wyhlen, Germany).

### Preparation of fibrinogen coated cover slips

Glass cover slips (24 × 50 mm) coated with 50 μl of fibrinogen solution (2,5 mg/ml, Sigma-Aldrich) that a sharp interface of the adhesion molecule was formed 10 mm away from the smaller edge of the cover slip [17]. The cover slip was placed in a humid environment (60 min at 37°C) to allow the protein to adhere to the glass surface. Coated cover slips were rinsed with 10 ml of 50 mmol/l phosphate buffered saline (pH 7.35, Serag-Wiessner, Germany) to remove the non-adherent fibrinogen and placed in the flow chamber. Fibrinogen density on the glass surfaces was calculated to be 0.13 μg/mm^2^. For the control experiments cover slips were coated with bovine serum albumin in final concentration of 5 μg/mm^2^. A specificity of binding of platelets to immobilized fibrinogen was tested in experiments with blood preincubated with Abciximab (c7E3, Centocor, Inc; 4 μg/mL, 10 min). c7E3 Fab is a chimerical human/mouse Fab fragment derived from the murine monoclonal 7E3 antibody that binds selectively to the GP IIb-IIIa.

### Flow chamber and laser-scan microscopy

The assessment of platelet adhesion rate onto fibrinogen coated glass cover slips was conducted in the rectangular flow chamber [17,18] under shear rate of 13 s^-1 ^50 s^-1 ^and 1500 s^-1^. Fibrinogen-coated glass cover slip with a flow path height of 50 μm, determined by silicon gasket, formed one side of flow chamber. Assembled flow chamber was filled by a phosphate buffered saline (pH 7.35). According to Newtonian fluid axiom, a shear stress is constant and dependent upon the flow rate (Perfusor, B. Braun, Meslingen, Germany). A shear rate of 13 s^-1 ^and 50 s^-1 ^mimics a venous wall shear rate and shear rate of 1500 s^-1 ^represents a shear rate of larger arteries as well as a high shear rate by moderate arterial stenosis [19]. One recent study indicated that a platelet function and a mechanism of adhesion and thrombus formation under these shear rates differ from each other [17,20]. A laser-scan microscope (Axiovert 100M, Carl-Zeiss, Jena, Germany) allowed real-time visualisation of labelled platelets during perfusion through the chamber. To assess time-course of platelet adhesion a series of images (five images per series, 0,7 s pro image) were made at 15 sec, 1 and 5 minutes. Image analysis was performed using the ImageJ software (version 1.26t, NIH, USA). This program allows evaluation of platelet-surface interaction, consecutive aggregation and evaluation of thrombus generation within the defined area of each image. A single frame image corresponded to the area of 980 × 980 μm. The blood was perfused over fibrinogen-coated cover slips as described above. The number of stable, attached platelets on the surface was calculated as number of platelets, which remain their initial adhering position in the first and second image (time frame of 0.7 sec). Platelets were considered to move on the surface when exhibiting a spatial displacement greater than one platelet diameter. To estimate motion, a series of 5 images (time frame 0.7 sec) at one time point were made. Using ImageJ software images were binarised and a threshold was applied to distinguish platelet from background. The first two consecutive frames in a series were superimposed using the logical AND function and the resulting image represented only the overlapping areas of single platelet at two different times.

### Calculations and statistics

Data in this study are given as mean values ± SD. The absolute fluorescence was expressed as arbitrary units (pixel units, AU) and represents sum of fluorescence of each thrombus or individual adherent platelet in one defined area. Only platelets, which show stable adherence during one image series, were taken into calculation. The platelet adhesion was calculated using a logic function of the applied software (ImageJ) and represented a stable platelet adhesion between the first and second image. A dynamic of platelets adhesion in function of time was defined as increase (or decrease) of the absolute fluorescence between the first and fifth minute of perfusion.

Differences between experimental groups were tested using Student's t-test (two-sided). Regression analyses were based on individual measurements using Spearman's rank correlation coefficient. Statistical analyses were performed using SPSS for Windows, version 6.0.1. *P*-value of less than 0.05 (two-sided) was used to indicate a significant difference.

To determine the contribution of the methodological variation (CV_analyt_) a representative sample was subdivided into six samples. Each sample was then separately processed and analyzed. From these individual measurements, CV_analyt _was calculated : Cv_analyt _= (CV_obs _^2 ^-CV_bio _^2^)^1/2^.

## Results

Clinical chemistry, haematology and haemostatic laboratory values in the blood obtained from 29 healthy blood donors were within the normal ranges. The average age was 44 ± 12 years and did not significantly differ between male and female (41 ± 10 vs. 46 ± 12 years, p > 0.05), respectively.

**Figure 1:**
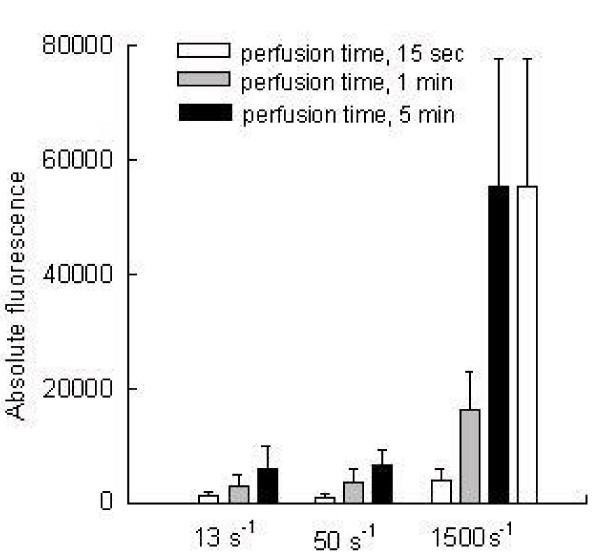
Platelet adhesion onto immobilized fibrinogen under different shear rate conditions with regard to the perfusion time (n = 29). 13 s^-1 ^and 50 s^-1 ^mimic venous flow and 1500 s^-1 ^arterial flow. Platelet adhesion between 15 sec and five min of perfusion increased five-fold at shear rate of 13 s^-1 ^and 14-fold at arterial shear rate of 1500 s^-1^. Per each subject 3 flow experiments were conducted (13 s^-1^, 50 s^-1 ^and 1500 s^-1^). Finally 87 perfusion experiments were conducted. At each time point of perfusion (15 sec, 1 min and 5 min) a stack of 5 images was collected and analyzed.

**Figure 2:**
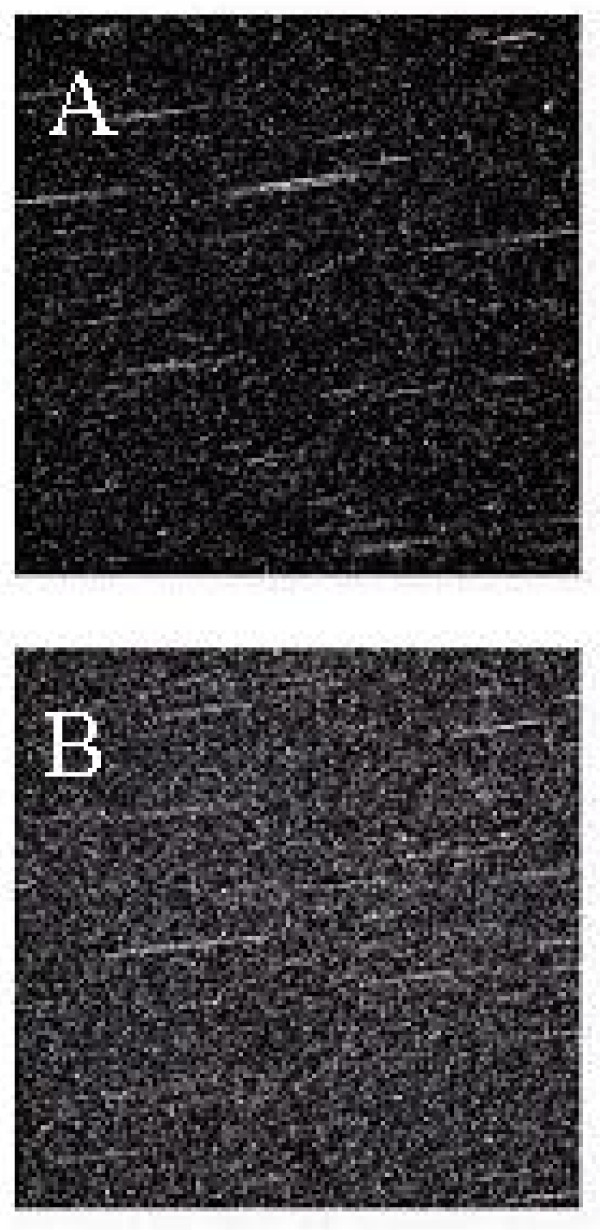
Microphotographs of platelets adhesion onto immobilized fibrinogen after 1 minutes (A) and 5 minutes (B) of perfusion at shear rate of 1500 s^-1^.

During the perfusion through the rectangular chamber, the platelet adhesion linearly increased with regard to the exposition time at each tested shear rate. From 15 sec to 5 min of perfusion the platelet adhesion rates were 5-fold (13 s^-1^), 6.8-fold (50 s^-1^) and 14.2-fold (1500 s^-1^), respectively. After five minutes of perfusion at 13 s^-1^, 50 s^-1 ^and 1500 s^-1^, averaged absolute fluorescence values were 6000 ± 3796, 6715 ± 2461 and 55170 ± 22530, respectively (Fig 1, Fig 2).

Taking into consideration individual and methodical variation, our experimental system was validated. Analytical variation (CV_analyt_), tested through 6 repeated measurements at 1500 s^-1 ^and 50 s^-1 ^(5 min of perfusion) of one individual sample, indicated that our analytical variation was 11% at 1500 s^-1 ^and 7% at 50 s^-1 ^respectively.

A specificity of binding of platelets to immobilized fibrinogen was tested bi-directionally with two additional experimental designs. In three experiments a blood was additionally incubated with Abciximab (4 μg/ml for 10 min), c7E3 fragment, which showed a high affinity to GPIIb-IIIa receptor. A perfusion (1500 s^-1^) of the preincubated blood over fibrinogen-coated cover slips omits to show significant platelet adherence (absolute fluorescence of stable adherent platelet: 168 U ± 35 U vs. 53000 U ± 19000 U at control experiments, p < 0.05). Similarly, in experiments with perfusion of labelled platelets in whole blood over BSA coated glass cover slips no significant adherence was found. The relationship between platelet adhesion and concentration of soluble plasma fibrinogen was not found (p > 0.05). Additional experiments, conducted with supplementation of soluble fibrinogen (up to 9 g/L), omitted to show any difference with regard to the untreated blood (data not present).

**Figure 3:**
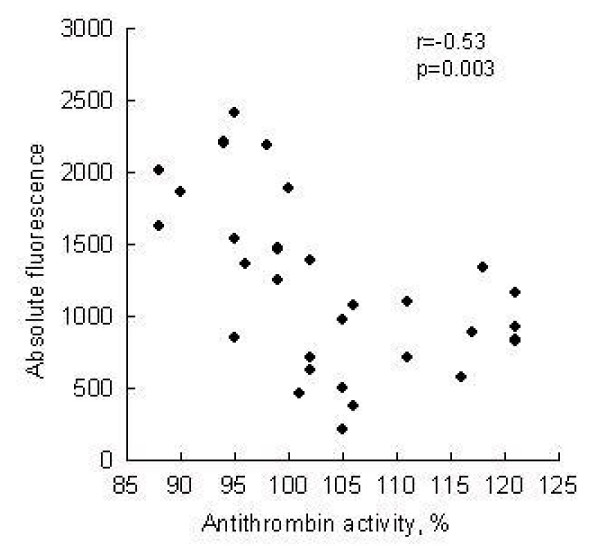
Plot of plasma antithrombin activity and platelet adhesion expressed as absolute fluorescence under low shear stress (n = 29, shear rate 13 s^-1^, 15 sec of perfusion). Spearman's rank correlation coefficient was -0.53, p < 0.05.

In each participant haemostatic laboratory parameters were measured including antithrombin plasma activity (Berichrom Anti-Thrombin III, Dade Behring, Marburg, Germany) and AT mean value was 103 ± 9%, ranged between 88% and 121%. Antithrombin plasma activity of each participant was carefully evaluated and compared to the platelet adhesion onto immobilized fibrinogen with regard to the shear rate and time of perfusion. Table 1. summarised relationship between plasma AT concentration and platelet adhesion at different shear rates. As it is evident from the Table 1., a significant inverse correlation between plasma AT concentration and platelet adhesion onto immobilized fibrinogen was observed at shear rates between 13 s^-1 ^and 50 s^-1 ^within one minute of perfusion (r = -0.53 and r = -0.72, p < 0.01). Fig. 3 represents a relationship between platelet adhesion and plasma AT activity at low shear conditions (13 s^-1^, 15 sec of perfusion).

**Table 1:** Relationship between platelet adhesion and plasma AT activity with regard to the perfusion time and shear rate

**Perfusion time**		**Shear rate, s^-1^**	
	13	50	1500
15 second	r = -0.53*	R = -0.72*	r = -0.11
1 minute	r = -0.26	R = -0.07	r = -0.07
5 minute	r = -0.34	R = -0.02	r = -0.05

*p < 0.05

Except for AT, no significant relationship was detected between platelet adhesion and other parameters at 13 s^-1 ^and 50 s^-1^. Parameters of plasmatic hemostasis as well as other biochemical parameters did not correlate with the platelet adhesion. Spearman's correlation coefficients between the platelet adhesion and examined variables were not significant (p > 0.05). In experiments conducted under arterial flow conditions (1500 s^-1^), no significant relationship was found between the platelet adhesion and plasma AT activity.

**Figure 4:**
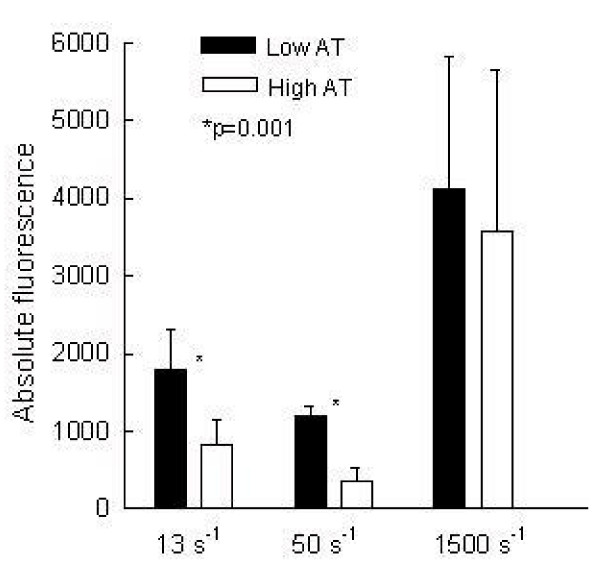
Platelet adhesion under different shear rates was selected according to the plasma antithromin activity of respected samples. Low activity = plasma AT activity <95% (n = 7) and high activity = plasma AT activity >105% (n = 13). Statistically significant difference in platelet adhesion with regard to the low and high AT activity was observed at 13 s^-1 ^and 50 s^-1 ^(p < 0.05). Similar trend was observed under arterial flow conditions but without statistical significance.

**Figure 5:**
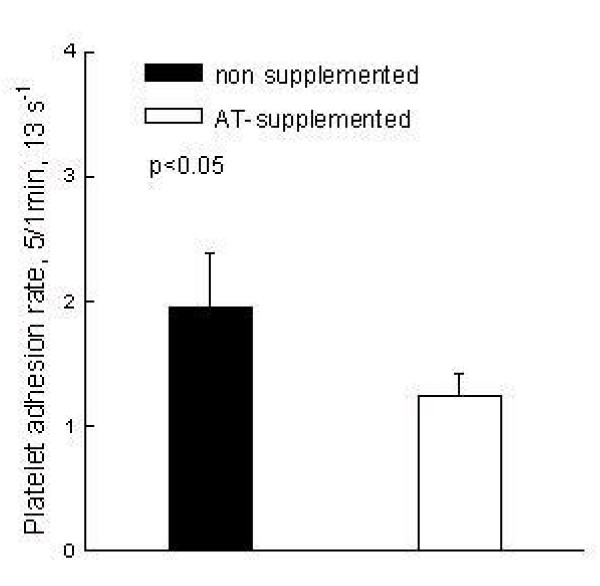
Anticoagulated blood (n = 8) was aliquoted and one aliquot was supplemented with AT (final activity 280%). Both aliquots were further perfused through rectangular flow chamber according to our experimental protocol. At low flow conditions (13 s^-1^) AT supplemented blood showed significantly lower platelet adhesion rate (65% of adhesion compared with non-supplemented blood, p < 0.05) between fifth and first minute.

The results of platelet adhesion were stratified according to plasma AT activity. Subjects with low and high AT plasma activity (<95% and >105%) were further statistically evaluated in the light of results of platelet adhesion. Fig 4. indicates that subjects with "low" plasma AT (92 ± 3%) have higher platelet adhesion at 13 s^-1 ^and 50 s^-1 ^comparing with subjects with "high" (117 ± 4%) AT plasma concentration, 1786 ± 516 vs. 823 ± 331 and 1190 ± 125 vs. 347 ± 186, respectively (p < 0.05). In the next set of experiments (n = 8) whole blood was aliquoted and one aliquot was preincubated 30 minutes with AT. After blood supplementation AT activity increased up to 280% comparing with 104 ± 9% in non-supplemented blood. The platelet adhesion rate (13 s^-1^) between the first and fifth minute after start of perfusion was 65% related to the adhesion rate of a non-supplemented blood, 1.25 ± 0.17 vs. 1.95 ± 0,4 p = 0.008, respectively, as shown in the Fig 5. Opposite to our expectation application of heparin as anticoagulant did not enhanced antiadhesion property of AT. The platelet adhesion rate was 2.42 ± 1.7.

## Discussion

The present study provides three major new results: firstly, a platelet adhesion onto immobilized fibrinogen is significantly influenced through the plasma antithrombin activity, secondly, this inhibitory AT effect was observed at low shear stress conditions in the initial phase within one minute, thirdly, a supraphysiological blood supplementation with AT significantly reduces platelet adhesion rate between the first and fifth minutes.

AT is a member of serpin super family of proteinase inhibitors and plays a crucial role in the maintenance of normal haemostasis. AT appeared at an early stage in the evolution of the vertebrate coagulation system. Andersen et al. [21] reported a full length AT sequence from Atlantic salmon fish, which appeared more than 450 million years ago. AT is not only a strong inhibitor of thrombin but also one effective inhibitor of factor IXa, Xa, XIa and XIIa. Kondo et al [22] reported significant inhibition activity of AT onto FVIIa. Described conventional and generally accepted biological role of AT in haemostasis also increased evidence about additional biological importance of AT [4,7-10,14].

The present study provides the first experimental evidence that the platelet adhesion onto immobilized fibrinogen is suppressed by AT in physiological and supraphysiological concentrations.

The first surprising result was significantly inversed correlation (p < 0.01) between platelet adhesion onto immobilized fibrinogen under low flow conditions (13 s^-1 ^and 50 s^-1^) and plasma antithrombin activity. Observations that platelet adhesion was significantly modulated through AT (even AT plasma activity of all participants was in physiological range, 88–121%) indicated a strong influence of AT plasma activity onto platelet adhesion. A comparison of platelet adhesion with belonging class (or subgroup) of AT activity has confirmed a great significance AThave on platelet adhesion under certain hemodynamic conditions. The platelet adhesion activity in subgroup with a high AT was 46% (average) lower, comparing to the subgroup with a low AT activity (p = 0.001). This significant influence of AT onto initial platelet adhesion (within first minute) and subsequently on the growth of thrombus in function of time was observed. The blood supplemented with AT (final activity 280%) showed averaged 36% lower platelet adhesion activity between the first and fifth minute comparing with non-treated blood. This information is of great importance since indicates AT influence on the thrombus growth.

The observed AT modulation of platelet adhesion activity is, according to our results, ligand, shear stress and time dependent. AT suppression of platelet adhesion was observed exclusively during the perfusion over immobilized fibrinogen under low flow conditions (shear rate between 13 s^-1 ^and 50 s^-1^). Under arterial shear condition (1500 s^-1^), no significant influence of AT was found. This data indicated that specific reaction between platelets and solid surface was simultaneously determined through the shear stress. According to the generally accepted theory, the platelet adhesion onto immobilized fibrinogen under low flow conditions (up to 20 dyn/cm^2^, equivalent 500 s^-1^) is exclusively modulated through GPIIb-IIIa (integrin α2bβ3) as specific fibrinogen receptor [19,23]. Application of Abciximab (specific inhibitor of GPIIb-IIIa) significantly reduces platelet adhesion onto fibrinogen and simultaneously abolishes AT modulated platelet adhesion. At the higher shear stress conditions, a stabile platelet adhesion onto immobilized fibrinogen is also, in time and dose dependent manner, modulated through von Willebrand Factor (vWF) [19,24]. The source of vWF can be plasma or platelet α-granules. We assume that AT also could have a certain influence on the platelet adhesion onto immobilized fibrinogen at high stress conditions but this influence could be mimicked through the significant vWF modulation.

Contrary to the fibrinogen, the platelet adhesion onto immobilized collagen (data not present) showed no correlation with plasma AT activity neither at venous nor arterial flow condition. A supplementation with AT and obtained supraphysiological AT plasma activity did not influence a platelet adhesion and consecutive thrombus growth onto immobilized collagen.

The absence of AT influence on the platelet adhesion onto immobilized collagen and shear stress dependent AT modulation of platelet adhesion onto immobilized fibrinogen indicated that AT modulation was directed through GPIIb-IIIa. Whether AT suppressed platelet adhesion is influenced through HPA-1 polymorphism (Leu33Pro polymorphism of integrin β3) could unfortunately not be evaluated due to low incidence of HPA-1b1b carrier in our study.

Mechanism by which AT suppresses a platelet adhesion is unclear. A ligand-specific and shear stress dependent modulation of platelet adhesion from blood already anticoagulated with PPACK (a strong thrombin inhibitor) as well as experiments conducted with heparin could indicated that AT suppressed platelet adhesion independent from thrombin inactivation. To test this hypothesis four experiments were conducted with heparin as anticoagulant. It is well known that the inhibition of thrombin through AT is by forming a 1:1 stoichiometric complex between the two components via a reactive site (arginine) – active centre (serine) interaction. This relatively slow interaction in the presence of heparin is dramatically accelerated [1,4]. Through the binding of heparin on the AT and consecutive change of AT configuration, a capability of AT to neutralise a clotting enzyme increased more than 1000-folds. Opposite to our expectation in experiments with heparinised blood, no significant influence of supplemented AT onto platelet adhesion rate comparing with the non-supplemented blood was not observed. This observation could indicated that AT act was independent from thrombin inactivation.

Uchiba et al. [7] indicated a promotional effect of AT onto prostacycline cellular production and a release of consecutive reduction of the platelet activation. We assume that this mechanism could not play significant role in our experimental system since allotment of activated platelets under low flow conditions was a very small and constant. Larsson et al. [4] demonstrated a remarkable anti-angiogenic effect of antithrombin in in-vivo animal model. The author explained anti-angiogenic effect through the observation that AT inhibits angiogenesis by impeding a focal adhesion (endothelial cells) formation and focal adhesion kinase activity. An incubation of the cells with AT was found to reduce fibroblast growth factor 2 induced focal adhesion kinase activity (FAK). FAK resides in the focal contact and its activity is induced by integrin-medited ligation of cells to the extra cellular matrix. FAK tyrosine phosphorylation is one of the early post-ligand-binding events mediated by integrins, and relies essentially on the functional β-subunit [25]. The exact mechanism by which AT perturbs FAK activation, remains to be determined. Being focused on an antiadhesion effect of AT, Yamashiro et al. showed that AT inhibited leukocyte rolling (tethering) along the retinal veins and that AT substitution significantly reduced P-selectin mRNA expression in the endothelial cells [26]. However, the mechanism by which AT exerts its suppressing effects on platelets adhesion is still unclear.

## Conclusion

A vessel-wall injury and consecutive fibrinogen deposition provides ideal environment and the stimulus for the initiation of clot formation [20,23,24]. An interaction between immobilized fibrinogen and platelets does not require previous platelet activation or conformational changes in platelet GP IIb-IIIa and has been shown to occur even in the presence of platelet inhibitors [23]. Since it has been widely believed that thrombus is formed in the areas of low shear and stagnation (e.g. recirculation zones) [18], our observations that AT significantly suppresses platelet adhesion onto immobilized fibrinogen under low flow condition might have a potential therapeutic significance and have to be further evaluated.

## Competing interests

The author(s) declare that they have no competing interests.

## Authors' contributions

RL: initiated the study, designed, coordinated and drafted the manuscript.

UK: participated in the design of the study.

VS: performed the flow experiments.

VT: performed the flow experiments.

RES: participated in the design and coordination of the study.

AV: participated in the design of the study and in the statistical analysis.
